# Heterogeneous short-term plasticity enables spectral separation of information in the neural spike train

**DOI:** 10.1186/1471-2202-13-S1-P98

**Published:** 2012-07-16

**Authors:** Felix Droste, Tilo Schwalger, Benjamin Lindner

**Affiliations:** 1 Bernstein Center for Computational Neuroscience, Berlin, 10115, Germany; 2 Institute for Physics, Humboldt-Universität zu Berlin, Berlin, 12489, Germany

## 

In order to understand how information is processed in the brain, it is vital to investigate how a single neuron responds to inputs that encode multiple signals. As each neuron receives input from many other neurons, such a situation is no exception but the norm. Previous studies have investigated how the transmission of one signal is influenced by others that can be considered background noise [1]. However, when looking at signal gating and processing, we also need to ask how the information content of more than one input signal is reflected in a neuron's output.

The interaction of signals is made complex not only by the nonlinearity of neuron dynamics, but also by short-term synaptic plasticity (STP) [2], which makes the amplitude of post-synaptic response dependent on the recent pre-synaptic spiking history. Synapses can exhibit qualitatively different kinds of STP. For instance, they can be predominantly facilitating or predominantly depressing. Previous studies have investigated the consequences of STP for information processing [3] and particularly pointed out that it can only lead to spectral filtering in the presence of noise or other signals [4,5].

In this study, we consider a scenario in which a neuron receives two stimuli through populations of purely facilitating and purely depressing synapses, respectively. Although such a setting is certainly an idealization, it resembles the difference in short-term plasticity of synaptic connections that parallel fibers and climbing fibers make to a Purkinje cell. Following the rate-coding paradigm, we model the input spike trains as inhomogeneous Poisson processes and use spectral measures such as the coherence to assess information throughput.

We find that STP leads to a spectral separation of information into high and low frequency bands. This spectral separation is based on the respective other signal acting as a kind of noise in the disfavored frequency band. Further, we show that the total information transfer about one signal can still benefit from the presence of the other signal through a form of stochastic resonance.
